# Letter to the Editor regarding “Systematization of Steps for Printing 3D Models of Orthopedic Deformities”

**DOI:** 10.1055/s-0045-1806816

**Published:** 2025-09-08

**Authors:** Amnuay Kleebayooon, Viroj Wiwanitkit

**Affiliations:** 1 Private academic consultant, Samraong, Oddar Meanchey, Cambodia.; 2 Saveetha Medical College and Hospitals, Saveetha Institute of Medical and Technical Sciences, Thandaram, Kancheepuram District, Tamil Nadu, India.

Dear Editor, “Systematization of Steps for Printing 3D Models of Orthopedic Deformities” 1 is an interesting article. The study described a method for creating patient-specific bone and joint models utilizing three-dimensional (3D) printing and preoperative computed tomography (CT) scans to generate a digital anatomical model. The method involves the use of digital imaging and communications in medicine (DICOM) for data segmentation in 3D imaging software, translating it to STL-file, and then printing with fused deposition modeling (FDM) technology. This method enables improved surgical planning and customization in orthopedic surgery.

While the study provides a foundation for 3D printing in orthopedic surgery, there are some technical challenges. First, conversion prevision varied based on original image quality and segmentation technique. Errors in these phases can result in inaccuracies in the final printed model, influencing the surgical outcome. Furthermore, the study did not address the choice of materials for 3D printing, which is critical for the mechanical qualities of the printed model, particularly when these are meant for preoperative usage to examine complex anatomical linkages. Printed materials differ in strength, flexibility, and biocompatibility, and should be thoroughly analyzed for stability.

Given the existing research and the proposed methodology, the emotional and psychological components of preoperative planning with 3D printed models are frequently disregarded. Customized models can provide tactile and visual feedback to both surgeons and patients, improving comprehension and communication regarding the surgical procedure and lowering anxiety. Furthermore, while the report describes the printing process, it does not adequately address the iterative nature of model customization, which may entail assembling a multidisciplinary team of surgeons, radiologists, and biomedical engineers to optimize the model based on feedback from surgical simulations, thereby increasing the accuracy and utility of the printed model.

Hospitals should establish protocols that incorporate the printing process and multidisciplinary collaboration. Adopting an interdisciplinary approach can drive model design innovation by providing feedback from many viewpoints, which can improve both model accuracy and usability in the operating room. Furthermore, the surgical team should be trained not only in the technology but also in how to use it in patients' contacts and communication.

To enhance the therapeutic application of 3D printing technology, educational programs should include hands-on workshops that cover both the technical and clinical elements of additive manufacturing, as well as the entire workflow, enabling orthopedic doctors to tailor technology to their needs. Using hands-on techniques, surgeons can experiment with various designs and printing parameters, generating a creative environment that may lead to novel solutions to surgical challenges. Future research could also explore merging 3D printing with augmented reality (AR) or virtual reality (VR).

These technologies could enable surgeons to study 3D models in a virtual space before operation, helping them to better understand intricate spatial anatomical linkages. Exploring this intersection could lead to significant breakthroughs in personalized medicine, which would precisely depict each patient's unique anatomy, resulting in better surgical outcomes. Furthermore, developing adaptive algorithms capable of automatically refining models based on surgeon feedback and postoperative outcomes has the potential to create a feedback loop that continuously increases model accuracy, eventually changing orthopedic practice.
